# Neurodegenerative Diseases: Regenerative Mechanisms and Novel Therapeutic Approaches

**DOI:** 10.3390/brainsci8090177

**Published:** 2018-09-15

**Authors:** Rashad Hussain, Hira Zubair, Sarah Pursell, Muhammad Shahab

**Affiliations:** 1 Center for Translational Neuromedicine, University of Rochester, NY 14642, USA; spursel2@u.rochester.edu; 2 Department of Animal Sciences, Quaid-i-Azam University, Islamabad 45320, Pakistan; hirazubair6@gmail.com

**Keywords:** neuroregeneration, mechanisms, therapeutics, neurogenesis, intra-cellular signaling

## Abstract

Regeneration refers to regrowth of tissue in the central nervous system. It includes generation of new neurons, glia, myelin, and synapses, as well as the regaining of essential functions: sensory, motor, emotional and cognitive abilities. Unfortunately, regeneration within the nervous system is very slow compared to other body systems. This relative slowness is attributed to increased vulnerability to irreversible cellular insults and the loss of function due to the very long lifespan of neurons, the stretch of cells and cytoplasm over several dozens of inches throughout the body, insufficiency of the tissue-level waste removal system, and minimal neural cell proliferation/self-renewal capacity. In this context, the current review summarized the most common features of major neurodegenerative disorders; their causes and consequences and proposed novel therapeutic approaches.

## 1. Introduction

Regeneration processes within the nervous system are referred to as neuroregeneration. It includes, but is not limited to, the generation of new neurons, axons, glia, and synapses. It was not considered possible until a couple of decades ago, when the discovery of neural precursor cells in the sub-ventricular zone (SVZ) and other regions shattered the dogma [1,2,3,4]. Neuroregeneration can also be defined as the progressive structural and functional recovery of the damaged nervous system over time. Damage to the central nervous system (CNS) is attributed to cell death, axonal regeneration failure, demyelination, and overall neuronal structural and functional deficits. All these conditions—partially or wholly, solitary or combined, genetic or acquired, known or unknown in origin—are manifested in specific neurological disorders, collectively termed as neurodegenerative disorders. These disorders jeopardize the normal functioning of the brain and lead to the progressive decline or even the complete loss of sensory, motor, and cognitive function. Examples include, but are not limited to Alzheimer’s disease (AD), Huntington’s disease (HD), Parkinson’s disease (PD), and multiple sclerosis (MS). 

Importantly, neurodegenerative diseases manifest in an abnormal buildup of proteins in the brain/tissue, i.e., β-amyloid in AD, misfolded Huntington protein in HD, aggregation of ubiquitinated proteins in amyotrophic lateral sclerosis [5], Tau and β-amyloid accumulation in MS plaques [6], α-synuclein accumulation in PD, and Tau neurofibrillary tangles in traumatic brain injuries [7]. Evidence suggests that the spread of misfolded protein from cell-to-cell significantly contributes to the progression of disease [8]. Moreover, in many cases, these misfolded proteins invade healthy brain tissue when two of these affected cells are placed together [8]. 

Given the widespread pervasiveness of potential neurodegeneration within the brain, many structures and regions are impaired. Consequently, synaptic insufficiency, massive cell death, inflammation, temporary or permanent loss of various bodily actions like coordinated motion (ataxias) and different cognitive skills like memory (dementia), decision-making skills, talking, breathing, and heart function, also become prominent features of neuropathology [9,10,11,12,13]. Whilst there are currently no cures for neurodegenerative diseases, particularly in advanced stages, developing therapeutic techniques is of the utmost importance to overcome physiological and cognitive deficits [14]. Recently, scientific literature has devoted countless studies to providing insight on novel therapy techniques to counteract and prevent the damaging effects of neurodegenerative diseases [15,16,17,18], particularly through ameliorating immune system and hormonal therapy, i.e., testosterone, estrogens, GH/IGF, etc. [19,20,21]. As research progresses, a greater understanding of the mechanisms that contribute to the progressive degeneration of neurons and their connections and the ultimate loss of cognitive and motor skills, could lead to more effective therapeutic techniques in the near future. 

## 2. Causes and Consequences of Neurodegeneration

All neurodegenerative diseases affect different regions of the brain, whilst exhibiting distinctive and apparent characteristics at the phenotypic level, i.e., progressive loss of sensory-motor and cognitive functions [22,23], but overall they share similar etiology at the cellular and molecular level [24,25,26]. Critical analysis of the similarities between these disorders offers the potential for therapeutic advancements, which could tackle many of these diseases simultaneously if we clearly understand the commonalities existing between various neurodegenerative disorders [27,28]. In this respect, neurodegeneration can be seen at different levels of neuronal circuitry, ranging from disturbance of intra-cellular protein molecules to inter-cellular disturbance of tissue and overall systems. 

Out of many different types of neurodegenerative diseases, Alzheimer’s (AD), Parkinson’s (PD), Huntington’s diseases (HD), and multiple sclerosis (MS), are the most commonly occurring forms. AD is the leading cause of dementia worldwide, causing the inability of an individual to perform everyday activities. An estimated 5.4 million Americans have AD, including approximately 200,000 aged <65 years, which comprises the younger-onset AD population. Statistics also show that every passing 68 seconds adds another patient of AD [29]. It starts as mild memory loss with symptoms worsening over time, and the affected person forgets how to perform basic daily activities like combing their hair and brushing their teeth. Over time, they become unable to recognize family members and need permanent care, which becomes a burden on society. The buildup of β-amyloid protein and intracellular aggregation of tau protein are the noxious etiological agents, which might trigger synaptopathies, glial inflammation, and eventual neuronal death in the cerebral cortex, sub-cortical regions, temporal and parietal lobes, and cingulate gyrus, observed in AD [30,31,32,33,34,35].

PD is characterized as a movement disorder, with an incidence of 0.3% in industrialized countries (Parkinson’s Association). It is caused by a decrease in brain dopamine (DA) levels. Death of DA producing neurons in substantia nigra is triggered by the intracellular accumulation of protein α-synuclein bound to ubiquitin complex [36]. These protein aggregates form cytoplasmic inclusions, commonly known as Lewy’s bodies, which play a significant role in familial and sporadic cases of Parkinson’s disease [37,38,39,40,41,42,43]. Symptoms begin as shaking of hands, arms, legs, and neck muscles. With time, severe ataxia develops, and the person fails to perform his everyday tasks [44,45].

Similarly, HD is also caused by the intracellular accumulation of aggregates of a mutant Huntington’s protein, resulting in brain cell apoptosis mainly in the striatum [46,47,48,49,50,51]. HD is a genetic disorder exhibiting movement problems and ataxia like PD after middle age, and progressive neuronal decline leads to the dementia-like AD [52,53,54].

AD, PD, and HD are primarily classified as proteinopathies, meaning they are associated with aggregation of misfolded proteins. AD and PD, both late-onset, are progressive diseases linked to the intracellular accumulation of toxic protein aggregates. Although, these diseases exhibit distinctive and apparent characteristics at the phenotypic level, at the subcellular level, they have a common etiology. In AD, neuronal death is seen in the amygdala, cortex, and hippocampus [9]. While in PD, substantia nigra shows neuronal loss leading to dopamine deficiency in the striatum [55]. 

In contrast to the above mentioned neuronal disorders, Multiple Sclerosis (MS) is a glial disorder. It involves massive damage to myelinated fibers through autoimmune reaction, causing axonal injury and further loss of neuronal communication mostly in the white matter tracts, the basal ganglia, and the brain stem [56,57,58,59,60,61,62,63]. This disease leads to a variety of physical, mental, and psychiatric problems [64,65,66,67]. In comparison to AD and PD, HD and MS are early in onset. AD, PD, and HD all have a strong genetic predisposition [68,69,70,71,72,73,74,75,76,77], where potentially the mutation is hereditarily passed down family lineage. In the AD, genetic heritability ranges from 49–79%, based on twin and family studies statistics [78]. In PD, 5–10% of those afflicted with the disease showed a mutation in different genes [79], leaving the individuals at greater risk to develop the symptoms. Similarly, as a purely a genetic disorder, HD is caused by trinucleotide repeat expansions in the Huntington protein [46]. Though unlike these genetically predisposed diseases, MS is not considered a hereditary disease. However, some genetic variations are believed to increase the risk of developing the disorder [80,81,82,83,84]. 

As discussed earlier, AD and PD share a phenotypic feature: Dementia. Dementia is an overarching term used to describe a group of symptoms, which result in a severe long-term decline in cognitive function significant enough to affect daily function. It can result from a number of complex disorders that damage the brain, including but not limited to AD, vascular dementia, frontotemporal dementia, dementia with Lewy bodies, and PD. Typical symptoms of dementia can include deficits in memory and language, impaired visuospatial skills, loss of executive function and attention, as well as behavioral disturbances [85]. Interestingly, autopsy analysis of a significant number of individuals diagnosed with AD or PD does not show the hallmark pathological feature of protein aggregate deposition [86,87]. Vascular dementia (caused by stroke) is also reported to be frequently occurring with AD, and this phenomenon could aggravate the symptoms of dementia [87]. Depending on the phenotypic features, vascular dementia can be misdiagnosed as AD or PD [88]. Overlapping pathological features of these diseases add further to diagnostic complexity. Data from studies investigating the link between neuropathology and molecular genetics have shown that phenotypic symptoms are not always highly associated with the underlying etiological changes, and they can be triggered by a number of other factors like experience, cognitive reserve, and epigenetics [89]. 

Focusing the neuropathological markers of dementia and defining it as a biological construct can lead to the discovery of novel genetic variants, the development of new peripheral biomarkers, as well as the identification of individuals at higher risk of the disease before the appearance of clinical symptoms [90]. This will also become an increasingly important issue as new drug treatments are developed [91].

## 3. Intra-Cellular Signaling Mechanisms

Compelling evidence indicates that the mTOR signaling pathway is involved in disease progress, aging, and regeneration. mTOR is a serine/threonine kinase, which in conjugation with other proteins makes two complexes: mTOR complex 1 (mTORC1) and mTOR complex 2 (mTORC2). Both complexes are phosphorylated by AKT dependent Pi3K, in which localization of both is solely cytoplasmic, but the field of operation is completely different. mTORC1 having raptor as its integral component promotes protein synthesis, ribosome biogenesis, proliferation, migration, and differentiation, by stimulating S6K1 and inhibiting 4EBP1 and elF4E. Whilst mTORC2 having rictor as its integral component promotes cell survival, cell cycle progression, and actin remodeling by its actions through PKC and SGK1. Studies suggest that mTOR promotes many of the processes which are impaired in HD, AD, PD, and MS [92,93,94]. However, the strategy of stimulating mTOR for a gain of function varies from disease-to-disease and case-by-case. 

For example, mTOR signaling is perturbed in PD [95], which is mainly attributed to a lack of L-DOPA resulting in the degeneration of the basal ganglia’s dopamine. However, treatment of L-DOPA nonspecifically activates mTOR; this over-activation produces symptoms of dyskinesia, which can be prevented by simultaneous treatment with rapamycin [96]. In mouse models of Parkinson’s disease, treatment of L-DOPA activates mTOR complex one, which is involved in neural synaptic rehabilitation directed toward the basal ganglia, proving to be a promising therapeutic approach for future clinical trials of PD [97]. 

Similarly, AD postmortem tissue analysis suggests a hyperactivation of mTOR/AKT/Pi3K, and increased levels of its downstream targets p70S6K and 4EBP1. This mTOR hyper-activation is significantly correlated with increases in the levels of beta-amyloid plaques [94]. Moreover, pre-clinical studies suggest that mTOR activation enhances Aβ generation and deposition by modulating amyloid precursor protein (APP) metabolism and upregulating β- and γ-secretases [98], while its inhibition by rapamycin ameliorates AD like conditions [93,99,100]. In HD, the mTOR pathway is hijacked by abnormally accumulated Huntington protein, which increases the mTORC1 activity [101], and results in massive damage possibly through inhibition of autophagy [102]. 

On the other hand, multiple sclerosis is a complex disorder where there is massive damage governed by inflammation [103,104], and in this disorder, mTOR activity is high and needs to be toned down [105,106,107]. Pre-clinical studies suggest that mTOR inactivation prevents the development of experimental autoimmune encephalomyelitis (EAE) in mice [108]. On the other hand, regeneration involving proliferation of oligodendrocyte, and subsequent differentiation clearly demands increased mTOR activity [109,110,111]. 

Beyond the mTOR complex implicated in neurodegenerative diseases, other signaling pathways have been found to be disrupted in disease pathology, which includes but is not limited to bone morphogenetic protein (BMP), mitogen activated protein kinase (MAPK), wnt/β catenin signaling, etc. [112,113]. Interruption of the BMP signaling pathway has been associated with failed synapse formation and maintenance in several neurodegenerative diseases. Decreased signaling has been noted in HD, while abnormally increased signaling has been found in MS [114]. In the case of Huntington patients, high levels of a protein thought down-regulate BMP signaling had been found in high concentrations [115]. It is thought that this excess is caused by the defected axonal transport. Axonal transport defects have been correlated with these BMP signaling deficiencies, such as in AD, even decades before official diagnosis [116]. Similarly, in AD patients, defected axonal transport has been observed along with the synaptic dysfunction in the inferior temporal gyrus, which has been associated with the loss of cognitive function [117]. Since the BMP signaling pathway maintains synapse function, the disruption of this pathway leads to an onslaught of negative consequences. BMP pathway is implicated in MS with increased signaling not only in mouse models of MS, but in MS patients [118]. Inhibition of the wnt/β-catenin pathway, which is essential for blood brain barrier formation, exacerbates MS like conditions in the animal model experimental autoimmune encephalomyelitis [113]. Overactivated MAPK signaling in AD increases neuronal apoptosis, as well as buildup of β-amyloid, through increased expression of β and γ secretases [113,119]; whilst it promotes neurogenesis in the mouse model of PD [120].

## 4. Current Treatment Paradigm

**Figure 1:**
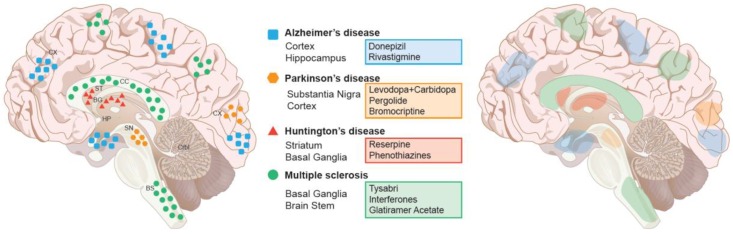
Major Neurodegenerative diseases, their associated regions, and current therapeutic interventions. Left panel: Brain disorders are color and shown in representative areas of the brain. Right panel: current pharmacological treatments and their areas of activity within the brain. Abbreviations: Basal ganglion (BG), Brain Stem (BS), Cerebellum (Crbl), Corpus callosum (CC), Cortex (Cx), Hippocampus (Hp), Striatum (St), Substantia Nigra (SN).

Current treatment strategies of neurodegenerative diseases merely target a small subset of the population and focus on symptomatic relief only, without altering disease progression (Summarized in Figure 1). This results in permanent disability or death of those afflicted. Presently, the Food and Drug Administration (FDA) has approved acetylcholine esterase inhibitors [Donepezil (Aricept), Rivastigmine (Exelon)], to be used as palliative treatment (see Figure 1 for action in different brain regions). 

These drugs reduce the symptoms and slow down the progression of the disease [121,122], but they are not useful in long-term treatment of the disease. PD is treated with Levodopa in combination with carbidopa (Sinemet), which crosses the blood-brain barrier and gets converted to dopamine after decarboxylation. It restores dopamine levels in the substantia nigra, and ameliorates all of the clinical features of Parkinsonism for the first few years, but loses efficacy on prolonged use [123]. Dopamine agonists [Pergolide (Permax), Bromocriptine (Parlodel)] are also in practice, but they cause a variety of adverse effects, including cardiovascular and endocrinological problems [124,125]. In contrast to PD, HD is caused by overactivity in dopaminergic nigrostriatal pathways. As a result, its treatment uses drugs that impair the dopaminergic transmission, either by depleting central monoamines [e.g., Reserpine (Serpadil)] or by blocking dopamine receptors [e.g., phenothiazines (Haldol, Trilafon)] [126]. MS is treated with many immuno-suppressers, which help to speed up recovery from relapse and slow down the progression of the disease. Therapies include MS relapse prevention by prednisone to reduce inflammation, Ocrelizumab for primary progressive MS, and a few other drugs for relapse re-emitting MS, including: beta-interferon (immunomodulatory), Ocrelizumab (neutralizing antibodies), glatiramer acetate, alemtuzumab and mitoxantrone (immuno-suppressor), Tysabri, and natalizumab, which provoke immune cells to enter into the brain [127,128,129,130] (Figure 1).

## 5. Future Strategies

Despite advancements in the fields of molecular biology, genetics, and pharmaceutical sciences, current biomedical science is a long way from the ultimate goal of identifying the risk factors, specific diagnostic tools, and appropriate strategies for the effective treatment of these neurodegenerative disorders. With an increasing burden on society, a need for newer treatment strategies is also in increasing demand. Current treatment approach mainly relies upon the survival of affected neurons and slowing the disease progression. This writing is based on some novel approaches (summarized in Table 1), which can promote the longevity of afflicted neurons and might slow down the degeneration process, improving the recovery rates.

### 5.1. Inhibiting and Disaggregating Protein Aggregates

A typical mammalian cell contains around 30,000 proteins, which are made up of linear chains of amino acids [131], and are responsible for all necessary biological actions. After transcription and translation, these proteins undergo post-translational modifications (mainly folding) to achieve the functional native form. Proper protein folding is under the control of the Protein Quality Control System (PQC) present inside the cell, which has evolved over an extended period [132]. Despite the activity of this system, many proteins fail to achieve normal functional folding state, because of multiple reasons like aging [133], oxidative stress [134], gene mutations [135], altered cellular temperature [136,137], and pH [138], etc.

Molecular chaperones act on these misfolded proteins to partially unfold them, which forms specific intermediates that might rearrange by themselves to build oligomeric aggregates that finally convert to amyloid fibrils [139,140]. In case of failure of chaperons, the lysosomal degradation system comes to the cellular rescue and targets these misfolded proteins. However, if this surveillance system also fails to cope with the situation, then the accumulation of these protein aggregates leads to the appearance of common neurodegenerative symptoms, caused by the amyloidosis of the CNS [141,142]. 

Short peptides, called synthetic mini-chaperons consisting of a recognition motif of a misfolded protein have shown some therapeutic potential in amyloid diseases. An example is heat shock protein (Hsp), a major molecular chaperone. Mammalian Hsps have been classified into families by their molecular weight, i.e., Hsp110, Hsp90, Hsp70, Hsp60, Hsp40, and Hsp27 [143,144]. Modification of the heat shock protein Hsp104, which upon interaction with other Hsps like Hsp110, Hsfp70, Hsp40, etc., very efficiently disaggregates non-desired proteins in yeast cells [145]. These peptides are designed so that they bind with the protein fibril at one end, but the other end does not contain any binding site [131], thus blocking the aggregation process. Moreover, modifications (N-methylation) of the amino acid backbone blocks intermolecular H-bonding, offering a steric hindrance in the formation of amyloid [146,147]. Promising effects of Hsp104 have been shown to dissolve various amyloid conformations and deposits of pre-amyloid oligomers. However, its application in humans requires a very high level dose in the present conformity, which seems utterly impractical. Similarly, several in-vivo and in-vitro studies have reported a neuroprotective role of Hsp70 and related compounds [148], by enhancing clearance of Aβ aggregates [149], and by restoring tau homeostasis [150]. This action of Hsp70 is attributable to upregulation of the insulin degradation enzyme (IDE) and TGF-β1expression [148]. Based on these molecular mechanisms, four major therapeutic strategies are proposed.

#### 5.1.1. Induction of Endogenous Hsp70 Production

Different compounds like galandamycin [151], geranylgeranylacetone [152], and celastrol [153], have been reported to induce endogenous Hsp70 production. These compounds give new leads for therapeutic strategy development related to neurodegenerative diseases.

#### 5.1.2. Application of Exogenous Hsp70

In a recent study, intranasal administration of Hsp70 was reported to improve cognitive deficits in AD like pathology in the mice model [154], possibly by reducing the production of reactive oxygen species [155]. However, this effect is not long lasting. Developing more effective dosage forms can be a potential therapeutic strategy for enhanced effect of exogenous Hsp70. 

#### 5.1.3. Constitutive Expression of Hsp70

Two cytosolic variants of Hsp70: Hsc70 and Hsp72, were previously reported to influence tau confirmation, degradation, and aggregation [156]. These variants are reported to have opposing effects on tau clearance. Hsp72 is reported to improve tau clearance, whilst Hsc70 is found to delay the clearance of tau protein aggregates [157]. Developing strategies to enhance Hsp72 expression selectively, and decrease Hsc70 expression, can prove to be an essential strategy to remove toxic protein aggregates and improve the pathological symptoms. 

#### 5.1.4. Inhibition of Hsp70 ATPase

Recently, selective Hsp70 ATPase inhibitor YM-08, has been found to reduce the level of pathogenic tau levels in brain cells, and retained its affinity in vitro [158]. Finding more chemicals like this can prove to be a crucial and highly selective therapeutic technique for AD. 

An alternative strategy is to increase endogenous production of Hsps. Studies show that cellular stress is the primary factor that upregulates Hsp, through activation of heat shock factor 1 (HSF-1). In healthy cells, Hsp most importantly, Hsp90 binds with HSF-1 and keeps it in an inactive form. While under stress, these repressors are occupied by misfolded proteins, thus leaving the HSF-1 un-repressed. Which leads to HSF-1 translocation to the nucleus, trimerization, phosphorylation at Ser4-419 residue and chromatin binding [159,160]. The exact details of this pathway and involvement of other protein complexes are not entirely known. However, a few studies show that Ral1 binding protein 1, tubulin, and P23 are very influential, and could be a potential drug target to increase endogenous production [161,162]. Collectively, these studies suggest a neuroprotective role of these molecular chaperones, but further efforts are needed to design a more efficient sequence to cope with dose associated problems, and the successful application in neurodegenerative disorders in humans to target β-Amyloid and Tau [145,163,164]. Late-onset neurodegenerative diseases, including PD and HD, are linked with the formation of intracellular toxic aggregates of different proteins. Therefore, understanding the factors that regulate the homeostatic levels of these proteins at synthesis and degradation stages is crucial [165].

After the aggregates have been formed, the normal function and structure of a protein can be restored by dissolving the aggregate. Protein disaggregates possess an innate property of breaking down these aggregates, and have shown therapeutic potential in the mitigation of the toxicity caused by these aggregates [166]. They dismantle self-templating amyloids and prion structures, which form neurotoxic oligomers. Different Hsp has shown potential in dissolving the protein aggregates, in metazoan and eukaryotic cells [167]. One example is eukaryotic Hsp104, an ATPase, but its activity is suboptimum. It can be therapeutically engineered to achieve an optimum level of therapeutic activity by induction of some mutations [167]. One variant of Hsp104 has shown therapeutic efficacy in dissolving α-synuclein aggregates in PD models [168]. 

HD is characterized by the polyQ, a residue that is known to cause protein misfolding in mHTT mutant Huntington [169]. Studies have found that Hsp70 targets the buildup of mHTT by binding to the proteins and halting further misfolding [170], preventing further neurotoxicity. Chaperones belonging to Hsp70 have also been found to specifically degrade polyQ proteins characteristic in HD, which has been observed both in vivo mice studies and in flies [171]. Stimulation of Hsp70 and the chaperones within that family by plant extracts, i.e., polyphenol curcumin or epigallocatechin gallate etc., can alleviate the cognitive and motor deficits that result from the neuronal death/damage caused by misfolded mHTT [172,173]. 

PD, on the other hand, is characterized by a buildup of α-synuclein misfolded protein aggregates. Hsp70, another molecular chaperone, identifies the α-synuclein by its hydrophobic region and binds to the protein, halting further misfolding and stimulating refolding [174]. Studies have shown that the absence of Hsp70 in disease models accelerates neurodegeneration in PD models, demonstrating the active role that this molecular chaperone plays in inhibiting further neuronal damage [175].

Certain Hsp, while still active in identifying and refolding protein aggregates, have also been found to assist in the buildup of misfolded proteins by protecting the neurotoxic proteins. Hsp90 has been found to support neurotoxic proteins often [176]. A pharmaceutical drug, geldanamycin has been found to inhibit protein buildup in PD dopaminergic murine models [177]. However, because geldanamycin cannot pass through the blood-brain barrier, other drugs must be developed to target HSF1, a transcription factor that stimulates the transcription of other HSPs that can effectively dissolve misfolded protein aggregates, as demonstrated in in-vitro studies.

The specialized roles of Hsp and their affiliated chaperones, have been found to specifically target certain types of protein aggregates in different neurodegenerative diseases. The identification of the specific Hsp and their correlated chaperones that provide the most significant assistance in targeting particular protein aggregates (mHTT, α-synuclein, etc.) for each neurodegenerative disorder and the development of drugs stimulating these heat shock proteins can prove beneficial for future therapeutic techniques [178]. Additionally, identifying Hsp that support neurotoxic buildup allows for targeted inhibition of these specific proteins, whilst stimulating the transcription of other useful Hsp and their relevant chaperones.

Combining protein disaggregation with protein degradation can be helpful in the elimination of toxic loss/gain of functional proteins, and reclamation of neuronal health.

### 5.2. Immuno-Modulation

Philosophically, neuroinflammation can be regarded as beneficial for the neuronal tissue to clean debris; but the scenario is more complicated when inflammatory mediators stay in the tissue longer than they are needed. It starts a vicious cycle of inflammation, which ultimately results in neuronal death and neurodegeneration. Elevated plasma and Cerebrospinal Fluid (CSF) concentrations of proinflammatory cytokines, such as IL-6, TNFα, IL-1β, IL-2, IL-6, and cyclooxygenase-1/2 have been found in PD, AD, and HD [179,180,181]. 

Microglia are present in the HD brain even before the onset of symptoms [182]. Detection of activated microglia and astrocytes have been found in HD patients up to 15 years before the symptomatic onset, and PET scans have indicated a correlation between microglia accumulation and HD pathology [183,184]. The activated microglia undergo morphological changes, such as an increased appearance of cytokines, specifically cytokine IL-6, in which increased levels are associated with increased severity of the functional decline in HD patients. Activated microglia, also undergo a morphological change in an ameboid fashion, meaning that the microglia take on an immune response with an increase of phagocytic activity, whilst also responding to damaged tissue. Therefore, detecting activated microglia even before the onset of HD symptoms is imperative in predicting the disorder’s severity [182,185,186]. Activated microglia can be classified as M1 and M2, based on the release of cytokines. Classically, M1 microglia release pro-inflammatory cytokines (IL-1β, IL-6, IL-8, etc.) and chemokines (CCL2, CCL20 and CXCL-1), whilst M2 microglia release anti-inflammatory cytokines and growth factors (IL-10, TGFβ, CD206 etc.); the latter initiates tissue repair and regeneration [187,188]. Post mortem studies suggest that HD patients have a significantly lower number of M2 microglia [188,189].

The central immune activation reflects a low-grade immune response in the periphery [190]. Furthermore, monocytes isolated from HD gene carriers, expressing the mutant Huntingtin protein, show hyperactivity to lipopolysaccharide stimulation [191]. Taken together, a hyperactive immune system proves to be an important feature of HD pathogenesis, and developing a strategy that deals with this hyperreactivity, using immunomodulators, is a potential disease-modifying avenue in HD treatment. Moreover, CD8+ and CD4+ peripheral lymphocytes have also been found in the substantia nigra of post mortem brains of PD patients [192]. Observational evaluation of large cohorts of patients, suggests that the use of non-steroidal anti-inflammatory drugs reduces the risk of PD [193].

Similarly, minocycline a potent anti-inflammatory drug which blocks activation of NAPDH oxidase and microglial activation, has also proven very effective in rodent models of PD [194]. Natural anti-inflammatory compounds like resveratrol [195,196], tanshinone [197,198], and silymarin [199], have shown therapeutic promise in animal models of PD, by downregulating the glial cell activation and pro-inflammatory cytokine release. Recently, sargramostim has shown therapeutic promise in a randomized, double-blind clinical trial [200]. In a recent in vitro study, laquinimod (an oral immunomodulator) demonstrated a dampening effect on the proinflammatory cytokine release from activated monocytes, and brings them down to base line activity [201]. 

Most of these inflammatory effects are mediated by microglia, where shutting down the microglia response altogether may even be more detrimental. It has been proposed that therapies should shift microglia response from pro-inflammatory M1 to the anti-inflammatory M2 Phase. The tone of microglia, i.e., pro-inflammatory to anti-inflammatory, can be managed by selectively improving the infiltration of regulatory T cells (Treg), producing IL-10 and BDNF [202,203].

Use of monoclonal antibodies against the α -synuclein, reduced the levels of protein propagation and demonstrated improved PD-like pathologies, ameliorated dopaminergic neuronal cell loss, and attenuated motor deficits in mouse models of the disease [204,205], whilst reducing amyloid formation [204,205]. It should also be noted that autoantibodies that can detect and degrade amyloidogenic proteins, like Ab, α-synuclein, and PrP are already present in the human serum [166,206]. 

Selective monoclonal antibodies to pathological proteins offer a novel avenue for the treatment of neurodegenerative diseases. Several monoclonal antibodies have been reported, which specifically bind to prefibrillar oligomers and not to amyloid fibrils, monomer, or natively folded proteins. Like the polyclonal antisera, the individual monoclonals recognize generic epitopes that do not depend on a specific linear amino acid sequence, but they display distinct preferences for different subsets of prefibrillar oligomers [207,208]. It is reported that autoantibodies that can detect and degrade amyloidogenic proteins, like Ab, α-synuclein, and PrP are already present in the human serum [166,206]. Table 2 highlights the antibodies currently undergoing clinical trials, for the treatment of proteinopathies.

### 5.3. Stimulating Autophagy

Autophagy, a cellular phenomenon responsible for removal of protein aggregates and dysfunctional mitochondria, is critical for the survival of long living, non-dividing cells, such as neurons and myocytes [209,210], as these cells are expected to accumulate unfolded or misfolded proteins in a significant amount because of their longer lifespan.

Initially, autophagy was thought to be inactive in neurons, as autophagosomes were found to be either completely absent from the neuronal cells or were found very rarely. Later, it was found that neuronal cells have a very effective lysosomal system, which is responsible for removal of autophagosomes rapidly, and inhibition of lysosomal activity leads to accumulation of autophagosomes [211]. It is not surprising that the mutation of gene-regulating autophagy causes neurodegenerative disorders, including AD, ALS, and PD [212]. Neurons have a very large expanse of axonal and dendritic cytoplasm. A long life span and limited regeneration make it very easy for neurons to gather large debris of dysfunctional organelles and waste proteins, which cannot be diluted because of limited cell division. Typically, phagocytosis starts with a change in phosphorylation of Unc51 by mTOR complex 11, followed by waste substance enwrapping by membrane-bound vacuole and fusion with lysosomes [212]. Various cellular stress signals, including decreased concentrations of amino acids, growth factors, hypoxia, and mechanical damages, suppress neuronal functions and plasticity through suppression of autophagy [213,214,215]. Flies and mice with autophagy gene (e.g., ATG) mutation, had a visible decline in neuronal health [216,217]. Specifically, the accumulation of aggregates of ubiquitin and faulty mitochondria was noticed in these cells [216,217], which are the hallmarks of neurodegeneration. These findings highlight the importance of autophagy in the maintenance of neuronal health.

Drug-induced autophagy is a promising regulatory approach to mediating the removal of abnormal proteins. Recently non-toxic, small molecules that induce autophagy in neurons have shown research potential as a therapeutic technique in animal models. Methyl-4-phenylpyridinium (MPP+) exposure has been found to induce apoptosis, in both in vivo and in vitro dopaminergic neurons in mouse Parkinson’s Models [218]. MPP+ is a neurotoxin that accumulates in dopaminergic neurons and organelles, with the toxin disrupting the complex I of the electron transport chain of mitochondria [219]. This inhibition induced by MPP+ leads to neuronal apoptosis. 

Similarly, latrepirdine, with the drug-patented name of Dimedbon, is an antihistamine that demonstrates promising results in mouse AD trials [220]. Latrepirdine regulates and protects the amyloid-β precursor protein (APP) associated with the buildup of Aβ proteins in Alzheimer’s. Meta-analysis showed a modest effect on behavior, but it did not show any beneficial effect on cognition in mild to moderate AD patients [221].

Whilst Lithium has been classically a major treatment of affective disorders, recent experimental and clinical studies have shown neuroprotective effects beneficial for the treatment of neurodegenerative pathologies, such as the regulation of autophagy and the synthesis of neurotrophic factors [222]. Drugs such as rapamycin and calcium channel blockers have also shown potential in stimulating the autophagic process in various studies done in Drosophila [223,224] and mouse models [102,225], where these showed an improved hepatic autophagic function in obese mice and increased clearance of mutant huntingtin protein. Rapamycin, an FDA approved drug, has been used therapeutically to reduce Aβ -induced cognitive deficits of Alzheimer’s [226]. It is also reported to reduce the risk of the AD in Type 2 Diabetes Mellitus (T2DM) rats by inhibiting the activation of AMPK-mTOR signaling pathway [227]. Mammalian rapamycin (mTOR) regulates protein synthesis and degradation in the mTOR complexes 1 and 2 [228]. Signaling of mTOR has been found to induce apoptotic pathways by forming autophagic vacuoles, ameliorating tau and Aβ in mouse models. 

Metformin has also shown beneficial effects in animal models of different neurodegenerative diseases like HD [229]. It induces the autophagic process by an AMPK-dependent mechanism. It also dephosphorylates neurofibrillary tangles of tau in AD clinical trials, by inducing protein phosphatase 2A (PP2A) activity in transgenic mice models [220]. As the major tau phosphatase in the brain, the induction of PP2A, which the drug Metformin targets, is vital to its therapeutic potential for neurodegenerative pathology [230]. Similarly, nilotinib has shown therapeutic promise in the improvement of PD profile by stimulating the autophagic process, inhibiting the tyrosine kinase activity in PD [231] and AD [232] mouse models. Continuing studies which highlight the further exploration of autophagy-improving chemicals can help in mitigating pervasive neurodegenerative symptoms (Summary in Table 3).

### 5.4. Clearance by Glymphatic System

Cellular debris and waste products are cleared from the body’s tissue by the lymphatic system. Unfortunately, the brain lacks a fully functional lymphatic system [233]. However, Nedergaard and co-workers discovered a parallel system in the brain, which drains neuronal metabolites by convective flow of cerebral spinal fluid through perivascular spaces [234,235,236]. The clearance pathway largely depends upon astrocyte-specific water channels AQP4; efficiency of this mechanism decreases with age, with an increase in AQP4 depolarization [237,238]. Deletion of these water channels which facilitate the convective fluid transport of CSF into the brain parenchyma and interstitial fluid back towards perivenous spaces [239], impair the normal influx/efflux of fluids. Moreover, this glia dependent lymphatic system analog—referred to as the Glymphatic System—is active only during sleep [234]. This identifies the importance of sleep, as well as the convective flow of CNS fluid, for proper and efficient removal of waste materials from the brain. 

More recent findings suggest that impairment of the glymphatic system is a potential risk factor for the development of neurodegenerative disorders, which are particularly associated with the buildup of abnormal protein as in AD [240,241,242]. Accumulation of β-amyloid protein in the intestinal space and along the vasculature, is a characteristic feature of AD. Importantly, its production and aggregation increase with age [243]. As β-amyloid accumulates, the glymphatic flow becomes disrupted, and waste clearance deteriorates.

Beyond just the waste clearance itself, the other functions of the glymphatic system have yet to be fully understood. Since the glymphatic system is thought to distribute lipids via the blood-brain barrier, it is possible that this system mediates growth factors and other neuromodulators which contribute to functional recovery in neurodegenerative diseases. Perhaps the glymphatic system distributes the molecules involved in neurogenesis, axonal growth, and neurotrophic factors. Thus, therapies to combat the negative domino effects of age on the glymphatic system, used in conjunction with the current understanding of the glymphatic system, should undergo further investigation. 

### 5.5. Neurogenesis and Neurotrophic Factors

Despite neurodegenerative damage to the brain, a degree of functional recovery is made possible through specific cellular and structural mechanisms that occur soon after the onset of an injury or cell death. Discoveries over the last couple of decades have refined the conception of the presence of neural stem cells within the brain. Neural stem cells are distributed throughout the brain, their presence and proliferation have convincingly been shown in the sub-ventricular zone, dentate gyrus of the hippocampus, and rostral migratory stream [244,245,246,247]. However, activation of these cells to participate in repair demands a very organized orchestration of events, which can only be visible during embryonic, early postnatal periods, and to a lesser extent during/after injury or brain insult. It involves active cell-to-cell communication, the presence of neurotrophic factors, and the activation of intra-cellular signaling mechanisms. 

Neurotrophic factors are imperative for recovery in neurodegenerative diseases. Often, neurodegenerative diseases result in dysregulation of neurotrophic factors [248,249,250,251], molecules that are specific to types of neurons and aid in neuron function and survival, specifically in proliferation, differentiation, and growth. Without functional regulation of these neurotrophic factors, neurons and glia tend to change shape and decrease in number, in affected regions of the brain [252,253,254,255,256]. Therefore, the mechanisms that regulate these neurotrophic factors must be galvanized with the onset of neurodegenerative diseases. 

Neurotrophic factors play two specific roles in the development and maintenance of the nervous system. During development, neurotrophic factors stimulate synaptic connections between neurons, and support axonal growth [257,258]. Then throughout adulthood, the neurotrophic factors maintain these synaptic connections and inhibit nerve cell apoptosis, regulating brain function [259,260]. Briefly, neurotrophic families consist of nerve growth factors (NGF), brain-derived neurotrophic factors (BDNF), ciliary neurotrophic factors (CNF), glial cell line-derived neurotrophic factors, Ephrins, endothelial growth factors (EGF) and transforming growth factors (TGF), neurotrophin 3 (NT3), and neurotrophin 4 (NT4). NGF and BDNF are particularly important in potential neurodegenerative disease therapies because, though they bind to different tyrosine receptor kinases, both utilize a similar pathway to promote cell survival through inhibition of apoptotic signals, and promote tissue growth by stimulating proliferation [261,262]. 

Since NGF and BDNF play important roles in the development and maintenance of neuron function and survival, the link between these neurotrophic factors and neurodegenerative diseases is unsurprising. Alterations of NGF and BDNF in the brain, and the disrupted binding of NGF and BDNF to their kinase receptors, are both linked to neurodegenerative diseases [263]. For example, studies have shown that a decreased level of NGF in Alzheimer’s patients leads to cellular death [264,265]. Similarly, studies have shown a decrease of BDNF in vulnerable brain areas, such as the substantia nigra in Parkinson’s patients [266,267]. In these areas of decreased neurotrophic expression, synaptic connections degenerate, leading to a cascade of synaptic transmission malfunction throughout this brain area without the proper maintenance that NGF and BDNF normally perform. As a result, neuron size and number begin to decrease, followed by a loss in neuronal function. 

Therefore, it seems only logical that the most straightforward therapeutic approach to combat this loss of neuronal function is to increase the neurotrophins in the given degenerating area of the brain; however, as therapeutic based research has exhibited, certain challenges arise when invasively delivering neurotrophins. Since neurotrophins are polar and relatively large molecules, they invariably cross the highly selective blood-brain barrier [268]. Furthermore, selectively controlling delivery of neurotrophins to only damaged neurons, rather than functional ones, is nearly impossible. 

Currently, research has provided researchers with several approaches to target neurotrophic deficits in the brain, despite these addressed challenges. These approaches include direct intracerebroventricular injection, viral vector-mediated gene delivery injection, and neurotrophin mimetics [263]. Whilst all these approaches exhibit potential, each approach comes with its share of side-effects. To avoid the negative side-effects of direct invasive injection into damaged brain areas, small molecules that mimic neurotrophins in its binding to specific tyrosine kinases is another viable option. These molecules are not neurotrophins themselves, but activate the same receptors, resulting in the same response as the binding of a neurotrophin to the receptor [61,269]. 

Other than neurotrophic factors, neural development in health and disease largely depends upon the presence of neurosteroids. The term was coined by French physiologist Etienne Baulieu, who demonstrated for the first time that the brain was capable of local synthesis of steroid hormones [270], to identify them from those produces by gonads separately. Being a very small molecular entity and non-polar nature, they cross the blood-brain barrier without any difficulty. Any change or deficiency within the neural tissue is readily compensated by circulating levels in the bloodstream. Deficiency in levels of these neurosteroids has been clinically validated in AD [271,272,273,274,275], PD [276,277], HD [278], and MS [279,280,281]; hormonal replacement therapy depicts beneficial effects in AD [276,282,283,284,285,286], PD [276,277,287,288,289], HD, and MS [256,290,291,292,293]. 

Stimulation of androgen receptors using neuro-active testosterone has beneficial influence on experimental autoimmune encephalomyelitis, a widely used disease model for the immune-mediated and inflammatory aspects of MS [294]. These neuroprotective effects are attributed to its immunomodulary and anti-inflammatory actions [295,296,297]. Recent clinical trials in relapse remitting MS patients, suggested an improved cognitive performance [256,292]. A detailed study of testosterone and its synthetic analogue in acute and chronic demyelination models, suggests that these effects are dependent upon the presence of neural androgen receptors [19]. Mice lacking an androgen receptor in neural tissue failed to recover and remyelinate their white matter tracts, even though they were administered with testosterone or its synthetic analog [19]. Similarly, the beneficial effects of testosterone and other neuroactive steroids (i.e., progesterone, estrogens etc.) have been documented for other neurological disorders like AD [276,282,283,284,285,286], PD [276,277,287,288,289], HD, and MS [256,290,291,292,293]. These neuro-regenerative effects of these neuroactive steroids most likely go through classical nuclear receptors [19,298,299,300,301,302]. 

Similarly, several studies showed the neuroprotective effect of estradiol in MS, AD, PD, and HD [263,303,304,305,306,307], whilst the lack of estrogens within the brain makes it more prone to neurodegenerative disorders. Administration of exogenous estradiol enhances neurogenesis by a mechanism that requires both α and β subtypes of estrogen receptors [308,309]. Additionally, estradiol promotes the migration of newly generated neurons towards the damaged brain regions [310]. Dual actions of estradiol: neuroprotective and anti-inflammatory [311,312], make it an ideal candidate for down-regulating the expression of pro-inflammatory cytokines, thus facilitating neuroprotection [313,314]. 

**Figure 2:**
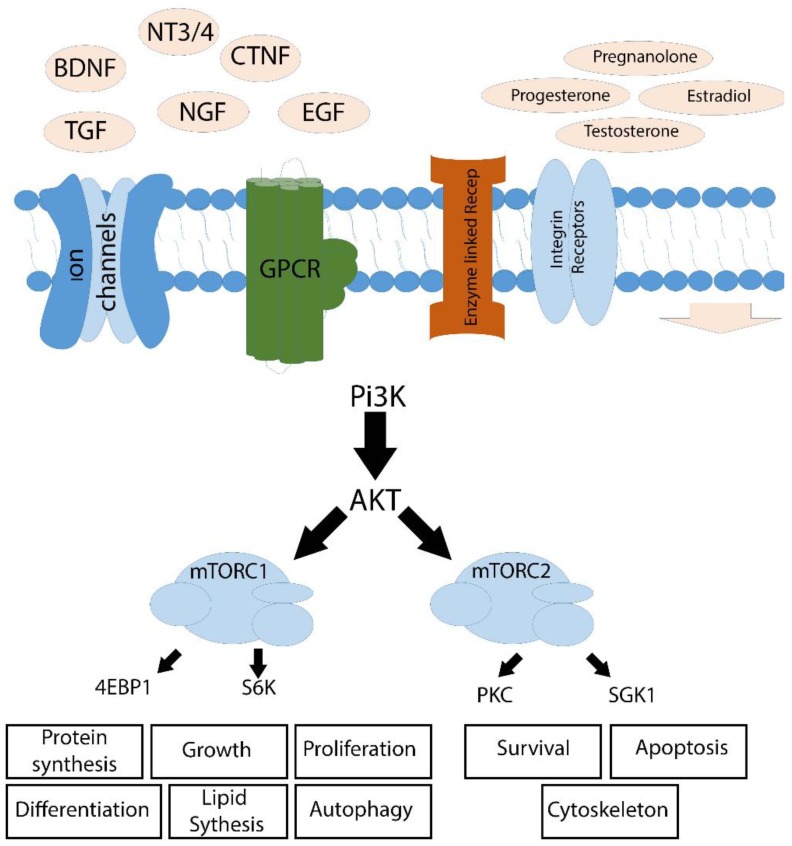
The interplay of neurotrophic factors, steroids, and intra-cellular signaling, in growth and differentiation of neural tissue. Cells sense different cues in the extracellular environment through membranous receptors, and these changes are communicated downstream through cascades of protein/cytoplasmic factor activation and inactivation. Many of these changes go through Protein kinase B or AKT, and mammalian target of rapamycin (mTOR), which further stimulate ribosomal proteins, i.e., 4EBP and S6 kinases, as well as PKC and SG1K. Stimulation of ribosomal proteins leads to protein synthesis, proliferation, growth, and differentiation; whilst PKC and SGK1 activation suppresses apoptotic pathways and improves survival. Abbreviations: BDNS (brain-derived neurotrophic factor), NT3 (Netrins 3), TGF (transforming growth factor), NGF (nerve growth factor), EGF (epithelial growth factor), CTNF (ciliary neurotrophic factor), GPCR (G protein-coupled receptor), Pi3K (phosphoinositide 3 kinase), Akt (protein kinase b), mTORC1 (mammalian target of rapamycin complex1), mTORC2 (mammalian target of rapamycin complex 2), 4EBP (eukaryotic initiation factor 4 binding protein), S6K (ribosomal protein S6 kinase), PKC (protein kinase c), SGK1 (glucocorticoid regulated kinase 1).

Presence and amelioration of the above mentioned neurotrophic alone are not enough. Regeneration still depends upon intracellular signaling. Typically, cells sense extra-cellular environmental changes through several membrane receptors (i.e., ligand-gated ion channels and receptors, enzyme-coupled receptors, GPCRs, and integrin receptors, etc.). These receptors are highly responsive to a very small change in extracellular environmental cues. Once a message is received, it is transferred across the membrane, and it involves a series/cascade of interactions between cytoplasmic proteins, including protein kinase B (AKT) [315,316,317], PI3K [318,319], Integrin-linked kinase (ILK) [320,321,322], microtubule-associated protein/ERK (p44/42) [323,324], mammalian target of rapamycin (mTOR) [325,326,327], and many more, which either translocate to the nucleus or activate chromatin binding proteins resulting in cell growth, proliferation, or differentiation (Figure 2).

### 5.6. Insulin and Neurodegeneration

In the central nervous system, insulin signaling promotes differentiation, proliferation, and neurite growth, and possesses neuroprotective and anti-apoptotic activity [328]. Insulin also modulates the structure and function of synapses, neurons, and neuronal circuits while playing a role in learning, cognition, and memory [328]. Neurochemical changes in neurodegenerative diseases may relate to abnormalities in glucose metabolism in vivo [329]. 

Brain insulin resistance partly drives cognitive impairment and AD in humans with peripheral insulin resistance diseases, including diabetes mellitus and metabolic syndromes [330,331]. Brain insulin resistance is also associated with increased levels of phospho-Tau and Aβ42 [332]. Experimental models have shown that brain insulin deficiency impairs learning and memory [333]. In early or intermediate stages of the AD, brain and CSF levels of insulin are found to be decreased [334], whilst Aβ42 and advanced glycation end-products are increased [334,335]. Insulin administration is found to improve working memory and cognition [336,337], and enhances Aβ42 clearance from the brain [337].

Activation of the insulin receptor (IR) leads to recruitment of insulin response substrate (IRS1 or 2) intracellularly [338,339], mediating the downstream signaling cascade. Insulin signaling is regulated by normal inhibition of IRS1 via MAPks [339]. The activity of these kinases can be modulated by extracellular factors like inflammatory mediators [340], oxidative stress [341], and Aβ oligomers [342,343]. Later, IRS-1 pSer616 and IRS-1 pSer636/639 were identified as putative biomarkers for brain insulin resistance in AD, and were reported to correlate positively with Aβ oligomer levels and negatively with cognitive function [344]. 

Dense representation of insulin receptors is reported on the dopaminergic neurons of the substantia nigra pars compacta [345]. Similarly, loss of insulin receptor immunoreactivity and messenger RNA in the substantia nigra pars compacta of patients with PD correlates with loss of tyrosine hydroxylase messenger RNA (i.e., the rate-limiting enzyme in dopamine synthesis) [346,347]. Abnormal glucose utilization has been noted in the brains of PD patients [348,349]. Abnormal glucose metabolism and increased frequency of T2DM has also been reported in patients with HD [350,351]. However, this link has not been studied intensively, and the underlying molecular mechanisms are not reported. 

Metformin, a common FDA approved the antidiabetic drug, is reported to inhibit glucose production by the liver and to increase the glucose uptake in peripheral tissues, thereby lowering the blood glucose levels [352,353]. Until now, only a few animal studies have assessed the effect of metformin on cognitive decline, and the results differ [354,355,356,357]. Whilst the results of clinical studies have shown a positive effect of metformin on cognition and neuropathological features [358,359,360,361], a greater understanding of metformin’s mechanism of action and development of more agents with similar activity may help to create novel therapeutic targets for these notorious diseases. 

### 5.7. Cholinergic System in AD

Recent data suggest that in contrast to early-onset AD, late-onset AD is a complex polygenic disease, which implicates atypical interaction between different molecular pathways. Clinically, the disease expression highlights the malfunction and ultimate collapse of both structural and neurochemical neuronal networks, including the cholinergic system [362]. Cholinergic synapses are ubiquitous in the human central nervous system. The significance of cholinergic transmission in higher brain functions like learning and memory is highlighted by the presence of high-density cholinergic synapses in the thalamus, striatum, limbic system, and neocortex [362]. 

Acetylcholine, an important neurotransmitter of the central nervous system, shows activity through the whole cortex, basal ganglia, and basal forebrain [363]. Human autopsy studies have shown that cholinergic loss is based on the degeneration of nucleus basalis of Meynert (NBM) cholinergic neurons, and of the axons they project to the cerebral cortex [364,365]. This cholinergic hypothesis of AD pathophysiology revolutionized the field of AD research, by carrying it from the traditional therapeutics to the contemporary concept of remedies based on synaptic neurotransmission. Three major discoveries led to the development of this hypothesis: (1) Discovery of depleted presynaptic cholinergic markers in the cerebral cortex [364,365]; (2) Discovery of the NBM in the basal forebrain as the major source of cortical cholinergic innervation, which undergoes severe degeneration in AD [366,367]; and (3) The demonstration that cholinergic antagonists weaken the memory, whilst agonists have the opposite effect [368]. This hypothesis received fascinating recognition when significant symptomatic improvement was induced by cholinesterase inhibitor therapy in AD patients [369], while anticholinergic drugs were reported to negatively affect human learning and memory [368,370,371]. 

Current therapeutic strategy in the management of AD, is based on the reclamation of cholinergic function by hindering the breakdown of acetylcholine [372,373] using cholinesterase inhibitors. Contemporary FDA-approved cholinesterase inhibitors in clinical practice are donepezil, rivastigmine, and galantamine. These compounds are reported to significantly improve cognition and some other behavioral features related to AD [373]. A meta-analysis of 26 studies of donepezil, rivastigmine, and galantamine reported a modest, but clinically significant, overall advantage of these drugs for stabilizing cognition, behavior, and global clinical change [374]. Early involvement of cholinergic system at preclinical stages of the disease was highlighted in different studies [375,376], which suggests that cholinomimetics can have a distinct role in disease prevention, along with other therapeutic agents. All this clinical and pathological data makes it very likely that cholinesterase inhibitors and discovery of other cholinomimetic agents can prove to be a very significant therapeutic intervention in the management of patients with AD.

## 6. Conclusions

In conclusion, the incidence of neurodegenerative disorders has been on the rise, and despite breakthrough discoveries, there remains an urgency from the patient’s perspective to search for and develop potential neuroprotective and neurorestorative therapeutics. Expansion of our understanding of intra, as well as inter, cellular signaling mechanisms both in health and disease, will greatly benefit our efforts to cure neurodegenerative disorders. Additionally, recapitulating the neuronal developmental paradigm in pathology and finding the means to create those conditions, including improved methods of drug delivery, would greatly enhance the chances of our success. Future research and clinical paradigms related to these notorious diseases may rely more heavily upon the ‘systems biology’ approach to these diseases, stressing the interaction of multiple factors such as genetic predisposition, stressors, inflammatory mechanisms, vascular insufficiency, dysregulation of protein aggregate formation, and clearance of neurofibrillary degeneration, cholinergic deficit, and other neurochemical anomalies. Therefore, despite substantial advances in the development of symptomatic treatments for neurodegenerative diseases, scientific efforts should not waiver, and perseverance is called for to attain this global goal.

## Figures and Tables

**Table 1:** Summary of novel treatment strategies for neurodegenerative diseases.

Strategy	Model	Drugs	Dose	Results	Reference
Inhibiting protein aggregates	Rat	iAβ5 (Chaperon)	200 nmol	Reduced size and number of cerebral amyloid plaques in AD	[377]
Disaggregating misfolded proteins	Mouse	Hsp104	-	Degradation of mutant HTT	[171]
	Mouse	Geldanamycin	1 or 10 µg/kg	Inhibition of HSP90 Induction of HSP70 Neuroprotection in MPTP induced PD	[177]
Immunomodulation	Mouse	Amyloid-β-Peptide	-	Reduction of Aβ deposits in the frontal cortex and hippocampus in HD model Improved cognitive function Reduced astrogliosis and disease progression	[378,379,380]
	Mouse	Resveratrol	50 mg/kg	Reduced glial activation in PD model Reduced ILs Improved TH expression	[195]
	Mouse	Tanshinone IIA	25 mg/kg	Reduced degeneration of nigrostriatal DA neurons Increased striatal DA content	[197]
	Mouse	Tanshinone I	10 mg/kg	Reduced expression of pro-inflammatory factors Improved motor function and striatal neurotransmitters	[198]
	Mouse	1H7, 5C1, 5D12	-	Reduced α-synuclein accumulation Reduced synaptic and axonal pathology in PD model Improved cognitive function	[204]
	Mouse	mAb47	-	Reduced α-synuclein accumulation in PD model Improved motor function	[205]
Induction of Autophagy	Drosophila	Rapamycin	1 µM	Enhanced clearance of pyroglutamine and polyalanine proteins Enhanced clearance of tau protein and decreased tau toxicity	[223]
	Verapamil	Mice	25 mg/kg	Suppressed hepatosteatosis Reduced obesity-induced cytosolic calcium in liver Restored autophagic flux	[225]
	Rapamycin ester (CCI-779)	Mice	20 mg/kg	Enhanced clearance of mutant huntingtin Improved behavioral tasks	[102]
	Metformin	Mice	2 mg/mL of drinking water	Prolonged survival time Decreased hindlimb clasping time	[229]
	Nilotinib	Mice	10 mg/kg	Reduced brain and peripheral α-synuclein and p-tau Improved immune profile	[231]
	Nilotinib	Mice	-	Increased β-amyloid clearance Reduced inflammation Improved immune profile	[232]

**Table 2:** Summary of monoclonal antibodies under clinical trials.

Antibody	IgG Subtype	Specificity	Dose	Reference
Bapineuzumab	IgG1 AAB-001 (humanized mouse 3D6)	Aβ 1–5 (helical, N-terminal D sensitive)	Phase I: 12-month 0.5, 1.5, or 5 mg/kg Phase II: 18-month 0.15, 0.5, 1, or 2 mg/kg Phase III: 18-month 0.5 mg/kg 1.0 mg/kg	[381,382]
Solanezumab	IgG1 (humanized mouse [265])	Aβ 16–26 accessible only on monomeric Aβ	400 mg every week for 76 weeks Phase III A4 400–1600 mg every 4 weeks for 240 weeks	[383,384,385]
LY3002813	IgG1 (humanized mouse mE8-IgG2a)	pE3-Aβ	0.1 mg/kg to 10 mg/kg, infused monthly up to four times, and a single subcutaneous injection against placebo for safety	[386,387]
Gantenerumab	b (RG1450, RO4909832) IgG1 (full human)	Aβ 2–5 (−9) + 23–25 binds with subnanomolar affinity to a conformational epitope on Aβ fibrils. It binds both N-terminal and central amino acids of Aβ	Phase III 225 mg SC	[388]
Crenezumab	IgG4 (humanized mouse MABT5102)	Aβ 13–24 (conformational epitopes) Binds fibrillar, oligomeric, and monomeric Aβ	Phase III up to 60 mg/kg SC (every 2 weeks) for at least 260 weeks	[389,390]
BAN2401	IgG1 (humanized mAb158)	recognizes Aβ protofibrils	Phase I: 2.5, 5 and 10 mg/kg Phase II: 2.5, 5 and 10 mg/kg	[391,392,393]
Aducanumab	b IgG1 (BIIB037/BART full human)	recognizes Aβ oligomer and fibrils		[394,395]

**Table 3:** Summary of pharmacological agents enhancing autophagy.

Drug Name	Type	Pathology	Model Type	Results	References
1-methyl-4-phenylpyridinium	Dopaminergic neurotoxin	Culture models of Parkinson’s	Mouse	induce buildup of autophagic vacuoles	[218,219]
Rapamycin	selective inhibitor of TORC1	Alzheimer’s Disease	Mouse	ameliorates Aβ and tau in AD mouse models	[226,228]
Latrepirdine	the stimulator of Atg5-dependent autophagy	Alzheimer’s Disease	Mouse	reduces Aβ in mouse models	[220]
Metformin	Protein phosphatase 2A agonist	Alzheimer’s Disease	Clinical Trials	Inhibits tau hyperphosphorylation in AD clinical trials	[220,230]
Lithium	UlK1 Kinase activator	Alzheimer’s Disease	Experimental/Clinical trials	AMPK activation and induces autophagic activation	[222]
